# Systematic review of the magnitude and case fatality ratio for severe maternal morbidity in sub-Saharan Africa between 1995 and 2010

**DOI:** 10.1186/1471-2393-11-65

**Published:** 2011-09-28

**Authors:** Dan K Kaye, Othman Kakaire, Michael O Osinde

**Affiliations:** 1 Department of Obstetrics and Gynecology, School of Medicine, Makerere University College of Health Sciences, P.O. Box 7072, Kampala, Uganda; 2 Department of Obstetrics and Gynecology, Jinja Regional Hospital, Jinja, Uganda

## Abstract

**Background:**

Analysis of severe maternal morbidity (maternal near misses) provides information on the quality of care. We assessed the prevalence/incidence of maternal near miss, maternal mortality and case fatality ratio through systematic review of studies on severe maternal morbidity in sub-Saharan Africa.

**Methods:**

We examined studies that reported prevalence/incidence of severe maternal morbidity (maternal near misses) during pregnancy, childbirth and postpartum period between 1996 and 2010. We evaluated the quality of studies (objectives, study design, population studied, setting and context, definition of severe acute obstetric morbidity and data collection instruments). We extracted data, using a pre-defined protocol and criteria, and estimated the prevalence or incidence of maternal near miss. The case-fatality ratios for reported maternal complications were estimated.

**Results:**

We identified 12 studies: six were cross-sectional, five were prospective and one was a retrospective review of medical records. There was variation in the setting: while some studies were health facility-based (at the national referral hospital, regional hospital or various district hospitals), others were community-based studies. The sample size varied from 557 women to 23,026. Different definitions and terminologies for maternal near miss included acute obstetric complications, severe life threatening obstetric complications and severe obstetric complications. The incidence/prevalence ratio and case-fatality ratio for maternal near misses ranged from 1.1%-10.1% and 3.1%-37.4% respectively. Ruptured uterus, sepsis, obstructed labor and hemorrhage were the commonest morbidities that were analyzed. The incidence/prevalence ratio of hemorrhage ranged from 0.06% to 3.05%, while the case fatality ratio for hemorrhage ranged from 2.8% to 27.3%. The prevalence/incidence ratio for sepsis ranged from 0.03% to 0.7%, while the case fatality ratio ranged from 0.0% to 72.7%.

**Conclusion:**

The incidence/prevalence ratio and case fatality ratio of maternal near misses are very high in studies from sub-Saharan Africa. Large differences exist between countries on the prevalence/incidence of maternal near misses. This could be due to different contexts/settings, variation in the criteria used to define the maternal near misses morbidity, or rigor used carrying out the study. Future research on maternal near misses should adopt the WHO recommendation on classification of maternal morbidity and mortality.

## Background

For every maternal death, there are close to 100 women with severe maternal morbidity referred to as maternal near misses [1-4]. Compared to maternal deaths audit, assessment of maternal near misses offers several advantages: maternal near misses are more common than maternal deaths; survivors can be interviewed, such that such review yields useful information on the pathways that lead to severe morbidity and death; and such assessment highlights the quality of obstetric care received [4-8]. The reported incidence of maternal near misses varies in different studies, and ranges from less than 1 per 1000 live births to 82 per 1000 live births [4,9-11]. It is important to note that different studies use different denominators (total births or live births).

The analysis of maternal deaths has long been used for the evaluation of quality of care, women's health and level of socio-economic development. The identification of cases of maternal near misses is an alternative to the investigation of maternal deaths when assessing the quality of obstetric care [2]. Conceptually, maternal near misses represent part of a continuum between extremes of good health and death [12]. On this continuum, pregnancy, labor or the puerperium may be perceived as uncomplicated, complicated, severely complicated or life threatening or fatal [5]. Indeed, from obstetric conditions, the woman may recover, become temporarily or permanently disabled, or die. The drawback in designating where a woman is positioned as a *maternal near miss *on this continuum lies in the definition of the threshold of severity above which morbidity qualifies to be a near miss. While this threshold is clear for some obstetric conditions or their management (for instance ruptured uterus managed by emergency hysterectomy, or severe postpartum hemorrhage requiring massive blood transfusion), it is may be uncertain or ambiguous for other conditions (such as sepsis). Secondly, the threshold above which an adverse obstetric event becomes life-threatening may be context specific. This is so because the probability of death from such complications depends not only on the woman's vulnerability to succumb to (or capacity to cope with) a given complication, but also on access to prompt and quality care [1,2,4,5]. The definitions used to identify a *maternal near miss *have to take the local context into consideration, and therefore health system factors. Three approaches have been proposed for definition of maternal near miss: utilization of clinical features (signs, symptoms or clinical entities such as eclampsia or uterine rupture) [13]; criteria of organ dysfunction [11], or criteria utilizing clinical management practices (such as admission to intensive care). Morbidity data is vital for health planners and policy makers who nee to know how many women need essential obstetric care. Morbidity data and case fatality ratios are essential and reliable indicators of the quality of obstetric care and the efficiency of the health systems, and therefore can supplement maternal mortality data. Maternal mortality ratios are difficult to use for evaluating the success of programmes (designing, monitoring and evaluating maternal mortality programmes) due to the difficulties in measurement. We assessed the prevalence/incidence of maternal near miss, morbidity, maternal mortality and case fatality ratio through systematic review of literature of studies from sub-Saharan Africa.

## Methods

### Data collection and inclusion criteria

We carried out a systematic review of the literature using the criteria described by Kranke [14] and Moher [15]. The search was conducted by a clinician and clinical epidemiologist. Medical and social science databases, including PubMed Medline, Popline, AIDSline, Scielo and Social Science Citation Index from 1996 to 2010 were searched for studies on life-threatening obstetric complications, severe acute maternal morbidity (maternal near misses) in sub-Saharan Africa, written in English, French or Portuguese languages. The key words used were "severe acute obstetric morbidity", "maternal mortality", "maternal deaths", "severe maternal morbidity, "severe acute maternal morbidity" or "near-miss maternal morbidity" limited to "sub-Saharan Africa", "females" and "adults". We also critically reviewed the reference list of these identified articles in an attempt to identify more articles. We analyzed studies which reported information (in pregnancy, childbirth or puerperium) on severe maternal morbidity. Maternal near miss as concept or paradigm began in the early 1990's in reference to women who survive severe acute obstetric complications. Our review attempts to highlight studies that utilized this concept or paradigm (irrespective of the terminology used to refer to these cases of severe acute maternal morbidity who would have died of pregnancy complications but somehow survived). Since it is very difficult to assess the quality of secondary data, all secondary data was excluded from this analysis. We included all cross-sectional, case control and prospective studies on severe maternal morbidity conducted in sub-Saharan Africa. Studies excluded from this analysis were those where there was no information on either morbidity or mortality and those where the specific obstetric conditions associated with mortality were not specified.

### Quality of methods and data abstraction

For the assessment of the study quality, a structured data collection form from the WHO systematic review of maternal morbidity and mortality [1] was used. The study quality was assessed by using the following criteria: description of study period, information about population characteristics, information about setting and context; information about eligible and lost subjects; definitions of conditions used (for maternal morbidity); forms of reporting data; information about using special efforts to capture all cases of severe morbidity or maternal deaths; limitations of the studies; and criteria used to address credibility and internal validity. Data on the incidence or prevalence of maternal near misses and case fatality was extracted. The prevalence or incidence ratio of maternal near misses was estimated as the total number of such events divided by the total number of participants in the particular study. Thus this incidence ratio represented the ratio of new cases with severe maternal morbidity divided by the total number of women at risk of such morbidity during the study period, while the prevalence ratio represented the ratio of all cases with severe maternal morbidity (during the study period) divided by the total number of women at risk of such morbidity. The case fatality ratio for a particular maternal near miss event was calculated as the proportion of fatal cases among the reported cases of the specified disease or obstetric condition.

**Figure 1:**
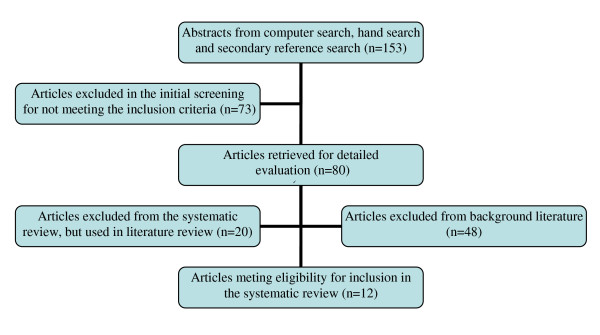
A flow chart showing the selection of articles included in the systematic review of the magnitude and case fatality ratio for severe maternal morbidity in sub-Saharan Africa between 1995 and 2010.

Figure 1 is a flow chart showing the selection of articles included in the systematic review of the magnitude and case fatality ratio for severe maternal morbidity in sub-Saharan Africa between 1995 and 2010. Initial review indentified 153 studies in articles whose titles and abstracts were scrutinized. Of these, 73 were excluded for several reasons that included: being qualitative studies, commentaries, opinion articles or ecological studies; being secondary data; or focusing on maternal mortality with no morbidity data. Eighty remaining studies (on any of the related descriptions of life-threatening obstetric complications) were eligible for inclusion in the systematic review. The full texts of these studies were retrieved and analyzed further.

Twenty studies were excluded from the systematic review, but used in literature review, while 48 studies were excluded entirely for lack of a clear definition of morbidity, unclear study design or unspecific data on the specific obstetric conditions that led to maternal mortality. Most of the 20 studies excluded from the systematic review, but formed background for the literature review, assessed a single or specific maternal morbidity (such as obstetric hemorrhage, abortion, ruptured uterus, sepsis or eclampsia), which was basis for exclusion from the systematic review. Finally, 12 studies were included in the systematic review. The authors of the articles that reported the studies used in this systematic review were contacted and requested to supply more information on the articles. We examined critically the definition used to identify maternal near misses, prevalence or incidence of maternal near miss and case fatality ratio from conditions that constituted maternal near misses.

## Results

We identified 12 studies which met the criteria for inclusion in the systematic review. Table 1 shows the characteristics of the setting, context, population studies and criteria used to identify maternal near misses. Seven studies were prospective studies, four were cross-sectional studies and one was a retrospective review of medical records or registers. The sample size varied from 557 women to over 40,000 women. The studies used different definitions for maternal near misses. While three studies used the management-based criteria for admission into the intensive care unit of a tertiary hospital, some studies used the diagnostic/clinical criteria for definition of maternal near misses. Nearly all the studies were local or geographical and not nationally or geographically representative of the regions where they were conducted. Secondly, some studies are health facility-based, so they is no base populations to which the results refer. Thirdly, different studies used different denominators (total births or live births) to derive the maternal mortality and morbidity that is presented

**Table 1:** Studies on near-miss obstetric morbidity in sub-Saharan Africa

Study	Country	Study design	Setting	Population	Sample size	Studied condition	Definition of near-miss morbidity	Comments
**West Africa**								
Prual et al, 2000 [28]	6 countries of West Africa	Cohort population-based prospective	Total population	Mixed (rural and urban)	20326	Haemorrhage, dystocia, sepsis, rupture of uterus, severe liver disorders, eclampsia, sepsis, cesarean section, thrombo-embolism, vesicovaginal fistula	Clinical diagnoses (such as haemorrhage with blood transfusion, sepsis - septicaemia, peritonitis or odorous vaginal discharge	Assessed quality of obstetric care.
Prual et al 1998 [29]	Niger	Cross-sectional	6 medical facilities	Unknown	4081	Severe obstetric morbidity: dystocia, uterine rupture, preeclampsia, eclampsia, haemorrhage, puerperal sepsis, infectious diseases and others	Based on clinical examination. Haemorrhage - hypovolemic shock requiring urgent blood transfusion	Severe complications from 28th week of gestation to42nd day post partum that would have resulted in death or disability without medical intervention.
De Bernis et al 2000 [31]	Senegal	Population- based cohort	2 regions	Urban	3777	Maternal morbidity: haemorrhage, hypertensive disorders, sepsis, obstructed labour, others	Clinical diagnosis and medical or obstetrical interventions.	Compare outcomes and management in two different regions.
Filippi et al 1998 [16]	Benin	Cross-sectional	Hospital	Unknown	4291	Near-misses: eclampsia, haemorrhage, dystocia, puerperal infections.	Not specified	Assessed quality of care for the near miss cases
Saizonou et al 2006 [34]	Benin	Prospective observational study	Health facility two teaching, two regional and three district hospitals	Mixed	557	All obstetric conditions including ectopic pregnancy	Criteria of signs and symptoms of obstetric complications	Assessed quality of care for the near miss cases
Oladapo et al 2005 [27]	Nigeria	Retrospective review of records	Health facilities	Mixed	1501	All obstetric conditions	Criteria of symptoms and signs and management-based criteria	Assessed quality of care. Calculated the maternal mortality index.
Mayi-Tsonga et al 2007[33]	Gabon	Prospective 6-month study	Health facility	Mixed	4350	All obstetric conditions as well as ectopic pregnancy and abortions	Criteria of symptoms and signs and management-based criteria	Assessed quality of care
**Southern Africa**								
Mantel et al 1998 [11]	South Africa	Cross-sectional prospective multicentre 2 year audit	Hospital	Unknown	13429	Near-misses: vascular, cardiac, immunological, coagulation, renal, respiratory dysfunctions, all ICU admissions, emergency hysterectomies, anaesthetic accidents	Clear definitions of haemorrhage, abortion complications, sepsis, pulmonary oedema and others	Based on organ dysfunction and management A near-miss describes a patient with an acute organ system dysfunction, which if not treated appropriately, could result in death.
Schoon 1999 South Africa [32]	South Africa	Incidence/prevalence, population-based cohort study	Region	Mixed	34100	Vascular, cardiac, immunological, coagulation, renal, respiratory dysfunctions, all ICU admissions, emergency hysterectomies, anaesthetic accidents	Clear definitions of haemorrhage, abortion complications, sepsis and others	Near-miss - all cases with organ dysfunction or organ failure during pregnancy of any gestation until 42 days after termination of pregnancy.
Vandecruys et al 2002 South Africa [35]	South Africa	Incidence/prevalence multicentric population-based prospective study	Region	Unknown	40006	Severe acute maternal morbidity (SAMM): hypertensive disorders, pregnancy related and not related sepsis, haemorrhage, abortions complications, embolism and others.	Clinical.	Audit of maternal death. Calculated the mortality index (MI)† defined as Maternal death divided by SAMM and maternal death. Compared data of different years.
**Eastern Africa**								
Kaye et al 2003, Uganda [18]	Uganda 2002-2003	Cross-sectional	Hospital	Women admitted in labor or pueperium	983	Severe life-threatening complications among emergency obstetric referrals	Severe acute morbidity from acute organ/system dysfunction,	Compared the utility of using organ/system dysfunction criteria to criteria of clinical signs/symptoms
Okong et al 2006, Uganda [6]	Uganda 2003-2005	Cross-sectional	Hospital	Women admitted in labor or puerperium	685	All obstetric conditions	Life-threatening complications	Assessed the quality of care and the three delays

Key: †The maternal mortality index is used to assess the quality of care in specific maternal conditions.

Table 2 shows the results of evaluation of the quality of the studies analyzed. Whereas some studies reported only the incidence/prevalence of maternal near-miss, others analyzed risk factors that may have contributed to the near miss as well as the quality of care at both the referring and referral units. In our assessment, most of the studies are of low methodological quality and rigor, much as multiple data sources were used. For the prospective studies, there was no information about loss to follow up, and for some studies, the population studied (to form the denominator for calculation of the prevalence or incidence ratio) was unclear.

**Table 2:** Assessment of the quality of studies on near miss obstetric morbidity in sub-Saharan Africa

Study	Location	Loss to follow-up (%)	Description of population characteri-stics	Data source	Description of study setting	Forms of reporting data	Case definition	Maternal deaths definition	Comments
Prual et al 2000 [28]	6 countries in West Africa	5.7%	Yes	Multiple source: interview, clinical examinations, records	Yes	Adjusted and standardized, used multivariate logistic regression	Clear	yes	special effort to capture all deaths
Prual et al 1998 [29]	Niger	-	Yes	Multiple source: medical records and interview	No	adjusted	No	No	Only definition of hemorrhage
Bernis et al 2000 [31]	Senegal	2.3%	Yes	Multiple source: case records and interview	Yes	adjusted and standardized, used multivariate logistic regression	Clear	yes	special effort to capture all deaths
Filippi et al 1998 [16]	Benin	-	No	Medical records	No	Crude estimates (frequencies and percentages)	No	No	special effort to capture all deaths
Oladapo et al 2005 [27]	Nigeria	-	Yes	Multiple source: case records and interview	Yes	Crude estimates (frequencies and percentages)	Clear	yes	special effort to capture all deaths
Mayi-Tsonga et al 2007 [33]	Gabon 2006	-	Yes	Multiple source: case records and interview	Yes	Crude estimates (frequencies and percentages)	Clear	yes	Special effort to capture all deaths
Mantel et al 1998 [	South Africa	-	No	Medical records and every morning audit meetings	Yes	Crude	Yes	yes	special effort to capture all deaths
Schoon 1999 [32]	South Africa	2.20%	Yes (Mixed)	Multiple: interview and case records	No	Standardised	Yes	yes	special effort to capture all deaths
Vandecruys et al 2002 [35]	South Africa	-	Yes	Multiple: daily audit meetings, case records	Yes	Adjusted and standardized	Yes	yes	special effort to capture all deaths
Kaye et al 2003, Uganda [18]	Uganda	-	Yes	Multiple: interview, investigations and case records	Yes	Crude estimates (frequencies and percentages)	Clear	Clear	special effort to capture all deaths
Okong et al 2006 [6] Uganda	Uganda	-	Yes	Multiple: interview, investigations and case records	Yes	Crude estimates (frequencies and percentages)	Clear	Clear	special effort to capture all deaths

Table 3 shows the incidence/prevalence ratio and case fatality for maternal near misses that was estimated from the studies. The prevalence/incidence ratio and the case fatality ratio range from 1.1%-8.3% and 3.1%-37.4% respectively. Wide variations exist between the studies within the same regions and within the same countries. While the variations could be dependent on the study design, the rigor in data collection or in the definition used for maternal morbidity (maternal near miss), the variation in case fatality ratios from countries where a similar protocol was used for the study suggests poor quality of obstetric care as a possible cause of high case fatality ratios.

**Table 3:** The incidence/prevalence*†*(1) (%) and case-fatality ratio‡ (2)% of severe maternal morbidity studies.

Study	Denominator	Severe maternal morbidity		Rupture of the uterus		*Haemorrhage		Sepsis		Eclampsia		Obstructed labor		Thrombo- embolism	
		1	2	1	2	1	2	1	2	1	2	1	2	1	2
Prual et al 1998, Niger	Live births	6.4	13.7	0.1	0	0.9	14.5	0.2	50	0.22	5.9	3.4	3.2	na	na
Mayi-Tsonga, gabon	Deliveries	3.15	Na	na	Na	na	Na	na	na	na	na	na	Na	na	na
Filippi et al 1998, Benin	Deliveries	8.2	8.5	na	na	2.3	9.2	0.4	21	1	4.7	4.5	3.6	na	na
Mantel et al 1998, South Africa	Deliveries	1.1	20.4	na	na	0.3	5.3	0.2	27.6	Na	na	na	Na	na	na
Schoon 1999, South Africa	Deliveries	5.3	37.4	na	na	0.06	27.3	0.03	72.7	Na	na	na	Na	0.02	83.3
Prual et al 2000, West Africa	Live births	6.6	3.1	0.1	30	3.05	2.8	0.09	18	0.19	18	2.05	0	0.02	50
Bernis et al 2000, Senegal	Deliveries	7.1	5.4	na	na	2.3	6.0	0.2	0	1.7	4.8	2.2	0	na	na
Vandecruys et al 2002, South Africa	Deliveries	1.1	20.0	na	na	0.83	Na	0.7	na	Na	na	Na	Na	na	100
Kaye et al 2003, Uganda	Pregnancies	10.1	1.7	3.7	11.8	6.2	6.3	2.9	3.6	8.8	3.4	20.1	2.0	na	na
Oladapo et al 2005, Nigeria	Deliveries	14.1	20.9	4.5	60.0	30.2	12.3	8.3	0.4	17.6	17.1	19.4	12.7	na	na
Okong et al 2006, Uganda	Pregnancies	33.4	na	na	Na	na	Na	na	na	na	na	na	na	na	na
Saizonou et al 2006, Benin	Pregnancies	26.7	na	na	Na	12.9	Na	3.4	na	24.9	na	8.7	na	na	na

Key: na Information not available

† The incidence/prevalence ratio was the number of near miss cases divided by the total number of patients

‡ The case fatality ratio was the total number of maternal deaths per obstetric condition divided by the total number of patients with the near miss obstetric condition

* hemorrhage includes both antepartum and postpartum hemorrhage

This systematic review shows that: 1) Different terminologies are used by different studies to describe women with severe acute maternal morbidity from complications of pregnancy, childbirth or the puerperium. 2) Different studies use different definitions of the maternal near miss. 3) Different studies collect and report differently the information on maternal near miss. 4) Few studies report on the specific causes of morbidity and their contribution to maternal deaths, making it difficult to compute case fatality ratios. Due to the differences in definition and terminology used, as well as nature of data collected, it is difficult to make comparison or reliably interpret data on maternal near miss and case fatality ratios from specific maternal complications (across the studies).

## Discussion

Severe acute maternal morbidity also known as "maternal near miss", is defined as "a very ill pregnant or recently woman who would have died had it not been that luck and good care was on her side" [11]. Our review shows that data on maternal near miss and case fatality ratios from specific obstetric complications is scarce in sub-Saharan Africa. Secondly, it is difficult to make comparisons as different terminologies are used to describe maternal near miss, there is no uniformity in definition of maternal near miss and subsequently, information on case fatality ratios from maternal near miss is difficult to interpret. In addition, our review indicates that different measures of the magnitude of maternal misses (prevalence or incidence) were reported by different studies. In real sense, the new cases of maternal near miss (referred to as incident cases) may not be different from all cases of severe morbidity reported during the study period (or prevalent cases) as both are directly related to complications that arise in the index pregnancy, labor or puerperium. This difference in measurement might make interpretation and comparability of these measures inaccurate.

From our attempt to harmonize the definition used in the few studies available, the incidence or prevalence ratio of maternal near misses) ranges from 1.1%-10.1% and case fatality ratio from 3.1%-37.4%, indicating wide variation in reported magnitude of the problem and case fatality ratios across the different countries. This review provides invaluable insights into the magnitude of maternal near misses, efficiency of maternal health services and quality of obstetric care in sub-Saharan Africa in the period 1995-2010. The review shows that obstructed labor, obstetric hemorrhage, ruptured uterus and sepsis (in descending order) are the commonest causes of maternal near misses.

Our review shows that in the 15 years (1995-2010), there was no standard definition of a maternal near miss. Therefore, different researchers used different definitions or a combination of different definitions of maternal near miss, namely the clinical/diagnostic criterion, the management-based criteria and the criteria of organ/system dysfunction. The advantage of the clinical approach in defining maternal near miss is that it is easy to interpret, appeals to both clinicians and non-clinicians, identified diagnoses tend to mirror the main causes of maternal death and this data is often routinely collected in medical records or hospital registers [5,7,8]. The limitation of using the clinical/diagnostic criteria is that different studies employ different cut-offs for what constitutes a maternal near miss. For instance, in studies from Benin [16,17], Uganda [6,18], Angola [19,20] and Burkina Faso [21], postpartum hemorrhage qualified as a maternal near miss, but additional data such as shock, massive transfusion or eventual hysterectomy contributed to the definition as a maternal near miss. For studies that used clinical signs and symptoms, hemorrhage, sepsis, hypertensive disorders, were the commonest definitions used. For studies that employed the management-based criteria for defining a maternal near miss, hysterectomy and admission to intensive care units were the commonest procedures employed [22,23]. In this criterion, indicators of severity of blood loss such as hypovolemia requiring massive blood transfusion, severe anaemia with hypotension (requiring intensive resuscitation) are used as proxy indicators for maternal near miss. This is dependent on the fact that utilization of high dependency obstetric care facilities or massive or prolonged resuscitation indicates a critically ill patient whether in pregnancy, labor or postpartum. Other management-based criteria include emergency peripartum hysterectomy and prolonged hospitalization for more than four days [24-30]. The limitation of this criterion is that it relies heavily on availability of management facilities. The justification of the organ-system dysfunction-based criteria, proposed by Mantel et al [11] is that women with organ/system dysfunction are likely to die unless adequate and prompt care is provided. For instance, obstetric haemorrhage constitutes a maternal near miss through vascular (hypovolemia), renal (oliguria) or coagulation dysfunction. Likewise, infection leads to maternal near miss in presence of respiratory, immunological or cerebral dysfunction. These may progress to multiple organ failure. The limitation of this criterion is that it relies highly on availability of diagnostic facilities to identify organ or system dysfunction. Many studies reviewed were audit studies [31-35] that employed a combination of criteria in definition of maternal near miss.

The differences in case fatality ratios are a reliable indicator of quality of care. For instance, in the prospective community-based cohort study by Prual et al [28] conducted in six countries, the methodology and questionnaires were the same in all areas. In this study, women pregnant woman had four contacts with the survey team: at inclusion, between 32 and 36 weeks of amenorrhoea, during delivery and 60 days postpartum. The overall incidence ratio of maternal near miss was in 1215 women or 6.17 cases per 100 live births (6.17%), but varied significantly between areas, from 3.01% in Bamako to 9.05% in Saint-Louis. The main direct causes of maternal near miss were: haemorrhage (3.05 per 100 live births); obstructed labor (2.05 per 100 live births), uterine rupture (0.12 per 100 live births); hypertensive disorders of pregnancy (0.64 per 100 live births), eclampsia (0.19 per 100 live births); and sepsis (0.09 per 100 live births). Case fatality ratios were high for sepsis (33.3%), uterine rupture (30.4%) and eclampsia (18.4%). For haemorahage, case fatality ratios were 1.9% and 3.7% for antepartum hemorrhage abruptio placenta respectively. The use of data collected on maternal near miss can therefore identify health system failures or priorities in maternal health care more rapidly than maternal deaths. However, as shown by this review, its routine use as an indicator is limited due to the lack of uniform criteria of identification of the cases.

Our review highlights the paucity of nationally or regionally representative studies on maternal mortality or maternal near misses, absence of a uniform definition of maternal near miss events and absence of uniformity in the terminology used to refer to women who have severe obstetric complications. The findings are in agreement with reports of inconsistencies in the way maternal deaths and severe maternal morbidity are classified or reported, as well as absence of standard definitions and criteria for identifying maternal near miss cases [1,7,36]. Severe maternal morbidity (maternal near miss) is a distinct disorder that should be assessed in addition to maternal deaths [37,38]. Our review also identifies scarcity of data on mortality from specific obstetric complications such that case fatality ratios for the specific conditions are impossible to estimate or compute. Due to the wide variation in identification of the cases, it is not possible to pool data and make a summary estimate for maternal near miss. Considering the variation in case identifications even within each category of identification criteria it is difficult to make comparisons as well. Nevertheless, it is evident that the prevalence/incidence of maternal near miss and case fatality ratios are high, which is an indication of poor quality of care.

Our review fulfils requirements in the consensus statement for reporting of systematic reviews of observational studies [39] in presenting a systematic process of data collection and interpretation. The implication of our findings is that there is urgent need to harmonize the terminology used in studies on maternal morbidity and maternal near misses as well as the denominators (population, total births or live births) used to estimate indicators. In agreement with other researchers [40], this will enable comparability across, times, settings, contexts and nations. Secondly future studies should report on the specific causes of maternal mortality (complications that cause maternal death) so that case fatality ratios can be estimated for given obstetric complications. Thirdly, there is urgent need to conduct longitudinal studies to assess maternal morbidity and mortality (using the WHO classification [1]). There is also urgent need to assess short-term and long-term impact of maternal near miss on reproductive health of women as well as that of their families and communities. The justification of the latter is the fact that occurrence of a maternal near miss event is a pointer to future reproductive morbidity among the survivors [41]. Indeed, from a study from Burkina Faso [42,43] and Benin [44], such survivors were found to have more unintended pregnancy, low contraceptive use and poor pregnancy outcomes compared to non-survivors. Likewise, in developed country contexts [45], obstetric complications significantly influence women's sexual health, wellbeing and subsequent fertility.

## Conclusion

Between sub-Saharan African countries, large differences exist on the magnitude of severe maternal morbidity (maternal near miss) in studies reported between 1996 and 2010. This could be due to different contexts/settings and variation in the criteria used to define the near miss morbidity, or rigor used carrying out the study. Researchers should adopt the new maternal death classification system to enable reliable comparisons to be made within and between countries and regions, as well as to enable identification of health system shortfalls that countries need to address.

## Competing interests

The authors declare that they have no competing interests.

## Authors' contributions

DKK conceptualized the study. DKK and OK designed the study protocol and designed the systematic review instruments. DKK, MOO and OK reviewed the literature. DKK wrote the first draft of the manuscript. DKK, MOO and OK contributed to revision of the subsequent draft manuscripts and approved the final version of manuscript.

## Pre-publication history

The pre-publication history for this paper can be accessed here:

http://www.biomedcentral.com/1471-2393/11/65/prepub
